# Induced Mitophagy Promotes Cell Cycle Re-Entry in Adult Cardiomyocytes

**DOI:** 10.3390/cells14120853

**Published:** 2025-06-06

**Authors:** Rafeeq P. H. Ahmed, Onur Kanisicak, Perwez Alam

**Affiliations:** 1 Department of Pathology and Laboratory Medicine, College of Medicine, University of Cincinnati, Cincinnati, OH 45267, USA; 2 Department of Emergency Medicine, The Ohio State University Wexner Medical Center, Columbus, OH 43210, USA; 3 Dorothy M. Davis Heart and Lung Research Institute, The Ohio State University Wexner Medical Center, Columbus, OH 43210, USA; 4 Department of Biomedical Sciences, University of Missouri, Columbia, MO 65211, USA

**Keywords:** adult cardiomyocyte, mitochondria, mitophagy, oxidative stress, cell cycle

## Abstract

**Background**: The limited regenerative capacity of adult mammalian cardiomyocytes (CMs) poses a significant challenge for cardiac repair following myocardial infarction. In contrast to adult mammals, CMs in zebrafish and newt hearts retain a lifelong capacity for proliferation and cardiac regeneration. Likewise, neonatal mice exhibit a brief postnatal period, during which CMs retain the ability to proliferate and contribute to myocardial repair, which markedly diminishes within the first week of life. Emerging evidence indicates that adult CM cell cycle progression is critically influenced by oxidative stress. Adult mammalian CMs possess a high mitochondrial content to meet their substantial energy demands. However, this also leads to elevated reactive oxygen species (ROS) production, resulting in DNA damage and subsequent cell cycle arrest. We hypothesize that reducing the mitochondrial content in adult CMs will mitigate ROS production, thereby facilitating cell cycle progression. **Methods**: Adult CMs were isolated from adult rats (≥12 weeks old). To induce mitophagy, adult CMs were transfected with parkin-expressing plasmid and then treated with carbonyl cyanide 3-chlorophenylhydrazone (CCCP), a mitochondrial protonophore, for 7 days. Post-treatment assessments included the quantification of adult CM proliferation, mitochondrial content, and ROS levels. **Results**: CCCP-treated adult CMs exhibited a significant increase in proliferation markers, including EdU incorporation, KI67, phospho-histone H3, and Aurora B. Furthermore, CCCP treatment significantly reduced the mitochondrial content, as evidenced by decreased MitoTracker, TMRM, and Tom20 staining compared to controls. This was accompanied by electron microscopy analysis, which showed a significant reduction in the mitochondrial number in the adult CM after CCCP treatment. Moreover, our results also demonstrate a marked reduction in oxidative stress, demonstrated by lower 123-dihydro-rhodamine (123-DHR), CellROX signals, and VDAC. **Conclusions**: Our findings demonstrate that CCCP-mediated mitochondrial depletion reduces oxidative stress and promotes cell cycle re-entry in adult CM. This study provides direct experimental evidence and substantiates the role of elevated mitochondria and ROS levels in adult CM cell cycle exit.

## 1. Introduction

The senescent nature of cardiomyocytes (CMs) is a major pathophysiological factor contributing to the limited regenerative and reparative capacity of the adult mammalian heart following injury [1]. In contrast to adult mammals, CMs in zebrafish and newt hearts retain a lifelong capacity for proliferation and cardiac regeneration [2,3,4]. Likewise, neonatal mice exhibit a brief postnatal period during which CMs retain the ability to proliferate and contribute to myocardial repair, which markedly diminishes within the first week of life [5]. Studies associate the senescent phenotype of adult CMs with elevated oxidative stress, compared to fetal or neonatal CM [6,7]. For instance, the hypoxic environment of the mammalian fetal heart, as well as some neonatal hearts that rely on glycolysis for energy production, show CM proliferation [8,9]. However, this proliferative capacity diminishes shortly after birth as CMs transition to an oxygen-rich environment and shift their energy metabolism toward oxidative phosphorylation to meet their increased energy demands [8,9,10,11]. Interestingly, the zebrafish heart, which resides in a comparatively hypoxic environment akin to the mammalian fetal heart, retains the lifelong ability to proliferate CMs and regenerate cardiac tissue after injury [12,13,14].

Adult CMs are mechanically active cells with exceptionally high energy demands, which are primarily met through oxidative phosphorylation and enhanced mitochondrial biogenesis. While oxidative phosphorylation generates approximately 18 times more ATP than glycolysis, it also produces elevated levels of reactive oxygen species (ROS) [15,16,17]. The detrimental effects of ROS in adult CMs include oxidative DNA damage, activation of the DNA damage response (DDR) pathway, and subsequent cell cycle arrest, all of which contribute to the pro-aging and senescent phenotype of these cells [6]. Metabolic reprogramming is a process in which cells shift their energy metabolism from oxidative phosphorylation to aerobic glycolysis, even in the presence of oxygen [18]. It has been observed across diverse cell types, including embryonic stem cells, retinal cells, macrophages, and cancer cells, which show proliferation [18,19,20]. Consistent with these findings, recent studies have underscored the therapeutic potential of hypoxia treatment in facilitating cardiac regeneration [21]. Hypoxia plays a critical role in the renewal of both neonatal and adult CMs by inhibiting oxidative DNA damage caused by aerobic respiration, thereby promoting the proliferation of pre-existing CMs [6]. Fate-mapping studies using hypoxia treatment have further demonstrated that proliferating CMs are predominantly in a hypoxic environment [22].

Furthermore, enforced mitophagy-targeted mitochondrial depletion has been shown to reduce the senescent phenotype in cultured fibroblasts [23]. Recent research has increasingly emphasized the critical role of mitophagy in the therapeutic management of various human diseases [24,25]. Mitophagy, an essential response to cellular stress, is critical in preserving mitochondrial network health by ensuring the timely removal of damaged mitochondria [25,26]. Meanwhile, impaired mitophagy contributes to the onset and progression of numerous diseases, including neurological, cardiovascular, pulmonary, hepatic, and renal disorders. Emerging data suggest that boosting mitophagy could serve as a promising therapeutic approach, aimed at restoring mitochondrial function, alleviating cellular stress, and improving clinical outcomes [24,27].

While studies highlight the functional relationship between the postnatal oxygen-rich environment, oxidative phosphorylation, elevated ROS production, and CM cell cycle arrest, the direct effect of induced mitophagy on adult CM cell cycle progression remains to be fully explored. We hypothesize that regulated mitophagy could enable adult CMs to re-enter the cell cycle. To test this, we employed the parkin-mediated ubiquitin–proteasome system to promote mitophagy, which selectively identifies and clears damaged mitochondria [23,28]. Carbonyl cyanide m-chlorophenyl hydrazone (CCCP), a mitochondrial depolarizing agent, was used to induce mitochondrial damage signaling. This activates parkin translocation to the mitochondria, where it ubiquitinates proteins for autophagic degradation, thereby reducing the adult CM mitochondrial mass. This system is widely used to study mitophagy and mitochondrial maintenance in various cellular contexts [24,29]. Our results provide a substantial link between an increased mitochondrial biomass, elevated ROS production, and the senescent phenotype of adult CMs. This insight is critical for understanding the factors contributing to adult CM senescence and cardiac aging, which is pivotal in strategizing novel and effective cardioprotective interventions to protect failing adult hearts.

## 2. Materials and Methods

### 2.1. Isolation and Culture of Adult CM

Adult CMs were isolated from 12-week-old Fisher rats using the Langendorff procedure, as previously described [30]. Briefly, the rat heart was excised and perfused with Krebs–Henseleit bicarbonate (KHB) buffer, followed by digestion buffer E until the heart became flaccid. The heart was then transferred from the Langendorff apparatus to a sterile laminar flow hood for the remaining steps of the procedure. The atrial portion was removed, and the ventricular portion was used for CM isolation. The CM suspension was filtered through a 100 µm cell strainer (BD Biosciences, Milpitas, CA, USA) and centrifuged at 300 rpm for 3 min. The supernatant was discarded, and the cells were re-suspended in 25 mL of buffer B, allowing them to settle by gravity. This step was repeated with an additional 25 mL of fresh buffer B. The isolated adult CMs were then cultured in Dulbecco’s Modified Eagle Medium (DMEM) (GE Healthcare, Chicago, IL, USA) supplemented with 10% fetal bovine serum (FBS) (Fisher Scientific, Waltham, MA, USA) and 5 mM of Penicillin/Streptomycin (Fisher Scientific, MA) at 37 °C in a 5% CO_2_ incubator for 24 h before transfection. The compositions of all solutions are provided in Table S1.

### 2.2. Induction of Mitophagy in Adult CMs via Parkin Transfection and CCCP Treatment

**Figure 1:**
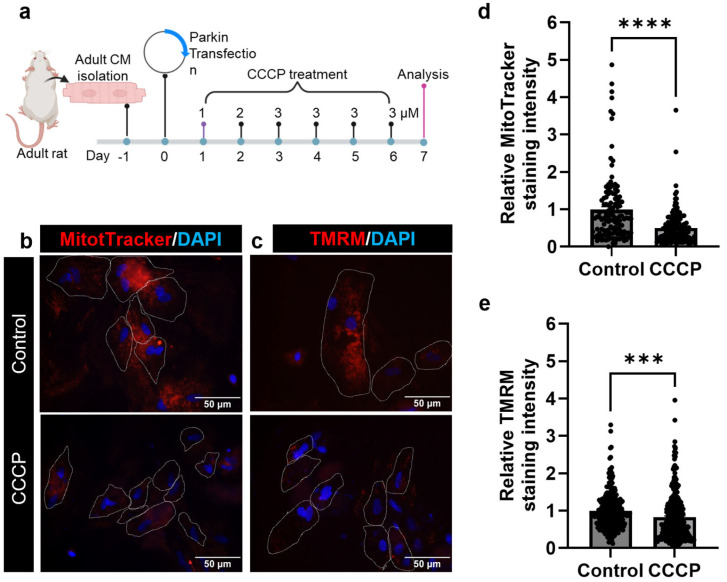
CCCP treatment decreases mitochondrial mass in adult CM. (**a**) Schematic overview of the in vitro experimental design, showing the isolation of adult CMs, parkin transfection, CCCP treatment, and subsequent proliferation and oxidative stress analysis. (**b**) Representative images of MitoTracker staining and (**c**) TMRM staining, demonstrating a decrease in mitochondrial mass in CCCP-treated adult CMs compared to control. Bar graphs (**d**,**e**) quantitatively show MitoTracker and TMRM staining intensities in CCCP-treated CMs *versus* control. Scale bar = 50 µm. Data are presented as mean ± SE; *** = *p* = 0.0003; **** = *p* < 0.0001. Statistical significance was determined using an unpaired *t*-test, with *p* ≤ 0.05 considered significant. A minimum of 30 cells per experimental group were analyzed per experiment, and all experiments were repeated at least three times. White dotted lines delineate the boundaries of individual CMs.

Mitophagy was induced in adult CMs using a parkin and CCCP-mediated system [23,31]. To overexpress parkin in adult CMs, we utilized a parkin-expressing plasmid (mCherry-parkin, or Parkin-GFP, Addgene, Watertown, MA, USA) [32]. Transfection was carried out using Lipofectamine 2000 reagent (Invitrogen, Watertown, MA, USA) according to the manufacturer’s protocol. Following transfection, cells were cultured for 24 h before CCCP or DMSO treatment. For CCCP treatment, adult CMs were treated with 1 µM of CCCP for the first day, 2 µM of CCCP for the second day, and 3 µM of CCCP for the third day. From day 4 to day 7, the cells were maintained in 3 µM of CCCP supplemented medium. The group transfected with the parkin plasmid and treated with CCCP was designated as the CCCP group. Meanwhile, the group transfected with the parkin plasmid and treated with DMSO served as the control. Post-transfection assays were conducted on day 7 after transfection, as illustrated in Figure 1a [33,34]. For EdU incorporation analysis, the culture media was supplemented with 0.5% EdU (Click-iT™ EdU Cell Proliferation Kit for Imaging, Alexa Fluor™ 555 dye; Invitrogen, Watertown, MA, USA; catalog # C10338) throughout the treatment period.

### 2.3. Immunofluorescence Analysis

On day 7 after parkin transfection, cells were fixed with 4% (*w*/*v*) paraformaldehyde and permeabilized through 15 min of incubation with 0.1% Triton X-100. To block nonspecific binding, cells were incubated with CAS-Block (Invitrogen, Camarillo, CA, USA) for 1 h at room temperature. Following blocking, cells were incubated overnight at 4 °C with specific primary antibodies (1:200 dilution) in CAS-Block. After primary antibody incubation, cells were incubated with fluorophore-conjugated secondary antibodies (1:200 dilution) for 1 h at room temperature. Nuclei were counterstained by incubating the cells with DAPI (4’,6-diamidino-2-phenylindole) for 20 min at room temperature. Troponin I (TnI) was used to specifically stain adult CMs. Images were captured using a widefield fluorescence microscope (Olympus; Center Valley, PA, USA) at varying magnifications. Details of the antibodies used are provided in Table S2.

### 2.4. Adult CM Proliferation

DNA synthesis in adult CMs was assessed using EdU staining. Adult CMs were cultured in the medium supplemented with EdU, allowing the incorporation of EdU into DNA during the S phase of the cell cycle. EdU incorporation was detected using the Click-iT™ EdU Cell Proliferation Kit (Alexa Fluor™ 555 dye; Invitrogen, Waltham, MA, USA, catalog # C10338), following the manufacturer’s protocol. In addition, specific phases of the cell cycle, including synthesis, mitosis, and cytokinesis, were analyzed through immunostaining with antibodies targeting markers such as Ki67 (BD Pharmingen, San Diego, CA, USA, catalog #550609), PH3 (Abcam, Cambridge, UK, catalog #ab51243), and Aurora B (Sigma-Aldrich, St. Louis, MO, USA, catalog #A5102), as described previously [33]. Immunofluorescence analysis was performed as described in the previous section. Adult CMs were specifically identified and labeled using Troponin I (TnI), and only dual-positive CMs were quantified to accurately and specifically assess CM proliferation. All experiments were performed in triplicate to ensure reproducibility.

### 2.5. Analysis of Mitochondrial Mass

The mitochondrial mass in adult CMs was assessed using Tetramethylrhodamine Methyl Ester (TMRM) and Mitotracker staining in live cells. Adult CMs were incubated with 100 nM of TMRM (Invitrogen, Waltham, MA, USA) and 200 nM of Mitotracker (Invitrogen, Waltham, MA, USA) at 37 °C for 30 min to evaluate the mitochondrial activity and distribution. Additionally, immunostaining for the mitochondria-specific protein Tom20 was performed to determine the mitochondrial content in the adult CM. The images were captured using the widefield fluorescence microscope, using fixed and exposure settings across all experimental groups to eliminate variability due to imaging conditions. A quantitative analysis of the fluorescence intensity was performed using ImageJ (Version 1.53e; NIH, Bethesda, MD, USA) for a minimum of 30 CMs per condition for each trial (*n* ≥ 3 trials). CMs were segmented manually and the mean fluorescence intensity for each marker was calculated on a per-cell basis. To account for inter-sample variation, the mean fluorescence intensity of the control group was used as a normalization reference. The intensities from individual cells in both the control and treatment groups were normalized to this baseline, yielding relative intensity values for comparative analysis. Data are presented as the mean relative fluorescence intensity ± standard error (SE).

### 2.6. Transmission Electron Microscopy

To complement the TMRM, Mitotracker, and Tom20 analyses, the mitochondrial biomass in adult CMs was further quantified through transmission electron microscopy (TEM). The samples were prepared and sectioned by the Pathology Research Core at Cincinnati Children’s Hospital, Cincinnati, OH, USA. TEM imaging was employed to provide high-resolution ultrastructural details of the mitochondria, enabling a comprehensive assessment of the mitochondrial morphology and abundance in both the treatment and control groups.

### 2.7. Analysis of Oxidative Stress

Oxidative stress was evaluated using the Dihydrorhodamine 123 (DHR123) assay (Life Technologies, Waltham, MA, USA, catalog# D-23806) and CellROX (Invitrogen, Waltham, MA, USA, catalog# C10444) analysis in live adult CMs. Mitochondrial oxidative stress was additionally assessed through voltage-dependent anion channel (VDAC; Cell Signaling, Danvers, MA, USA, catalog# 4661P) staining in fixed adult CMs. DHR123 is a reactive oxygen species (ROS) indicator that passively diffuses across the cell membrane, where it is oxidized in the mitochondria to form the cationic rhodamine 123, which emits green fluorescence. Adult CMs were incubated with 5 µM of DHR123 (Life Technologies) at 37 °C for 30 min to detect ROS levels. CellROX analysis was performed by incubating adult CMs with CellROX Green for 30 min at 37 °C. Upon oxidation, CellROX exhibits increased fluorescence, indicating the ROS level. The immunostaining for the VDAC was performed as the procedure described previously. CMs were double stained for the TroponinI (TnI), and only TnI-positive cells were included in the analysis. As previously mentioned, the images were captured using the widefield fluorescence microscope, using fixed and exposure settings across all experimental groups to eliminate variability due to the imaging conditions. A quantitative analysis of the fluorescence intensity was performed using ImageJ (Version 1.53e; NIH, Bethesda, MD, USA) for a minimum of 30 CMs per condition for each trial (*n* ≥ 3 trials). CMs were segmented manually, and the mean fluorescence intensity for each marker was calculated on a per-cell basis. To account for inter-sample variation, the mean fluorescence intensity of the control group was used as a normalization reference. The intensities from individual cells in both control and treatment groups were normalized to this baseline, yielding relative intensity values for comparative analysis. Data are presented as mean relative fluorescence intensity ± standard error (SE).

### 2.8. Statistical Analysis

All data are presented as mean + SE. The student’s *t*-test was applied for comparisons between the two groups. More than two groups were compared through one-way ANOVA (GraphPad Prism 10.2.0), and the *p* value was adjusted through Tukey’s multiple comparisons test. Significance was defined as *p* ≤ 0.05. All the experiments were performed in triplicate and repeated at least twice.

## 3. Results

### 3.1. Mitophagy Regulator Screening Reveals CCCP as a Potent Inducer of Adult Cardiomyocyte Proliferation

To investigate the role of mitophagy in adult CM proliferation, we screened several known mitophagy regulators, including rapamycin, chloramphenicol, and carbonyl cyanide m-chlorophenylhydrazone (CCCP). No significant increase in adult CM proliferation was observed at any concentration of rapamycin (Supplementary Figure S1). Chloramphenicol treatment induced a moderate increase in ACM proliferation, but only at 2 μM (Supplementary Figure S2). Furthermore, co-treatment with rapamycin (2 μM) and chloramphenicol did not result in a significant increase in EdU-positive CMs compared to individual treatments (Supplementary Figure S3). Interestingly, CCCP at a 2–4 μM concentration induced the most robust ACM proliferation (Supplementary Figure S4a). The highest level of cell cycle activation was observed at 3 μM of CCCP. However, co-treatment with CCCP (3 μM) and chloramphenicol (2 μM) did not produce an additive effect on proliferation (Supplementary Figure S4b). Given its robust effect on adult cardiomyocyte proliferation, CCCP was selected for further analysis.

### 3.2. CCCP Treatment Decreases Mitochondria in Adult Cardiomyocyte

**Figure 2:**
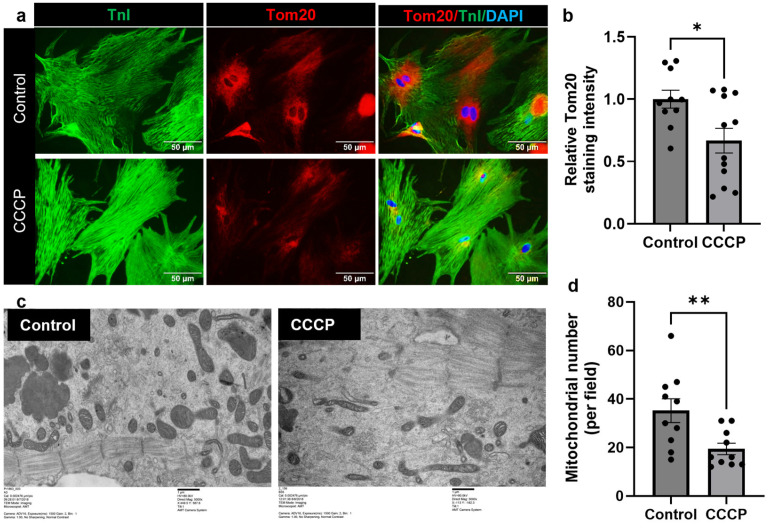
CCCP treatment induces mitophagy in adult CM. (**a**,**b**) Representative immunofluorescent images of Tom20 and corresponding bar graph, showing a reduction in mitochondrial mass following CCCP treatment compared to the control. A minimum of 10 images per group were analyzed from three independent experiments. (**c**,**d**) Representative TEM images revealed a decrease in the mitochondrial number in adult CMs treated with CCCP compared to the control, with 10 images analyzed per group. Scale bar = 50 µm or as indicated. Results are presented as mean ± SE; * = *p* ≤ 0.05; ** = *p* = 0.0096. Statistical significance was determined using an unpaired *t*-test, with *p* ≤ 0.05 considered statistically significant.

To comprehensively assess CCCP-induced mitophagy, we employed MitoTracker and TMRM for live cell analysis, along with Tom20 for an analysis of fixed cells. We observed a significant reduction in the fluorescent intensity of the mitochondria-specific marker MitoTracker (0.501 ± 0.034) in the CCCP-treated group compared to controls (1 ± 0.08; *p* < 0.0001) (Figure 1b,d). Similarly, the intensity of TMRM fluorescence was decreased (0.83 ± 0.04) in the CCCP-treated group relative to the control (1 ± 0.03; *p* < 0.003) (Figure 1c,e). Both MitoTracker and TMRM are membrane potential-dependent markers and label only functional mitochondria. To evaluate the total mitochondrial mass in CCCP-treated adult CMs, we conducted an immunofluorescent analysis using Tom20. Importantly, Tom20 analysis corroborated the results observed with MitoTracker and TMRM, showing a significant decrease in fluorescent intensity (0.67 ± 0.11) in the CCCP-treated group compared to the control (1 ± 0.07; *p* = 0.016) (Figure 2a,b). For the clearer visualization and better separation of fluorescence channels, representative images for Tom20 staining are also rendered in a magenta/cyan format (Supplementary Figure S5). MitoTracker tracks mitochondrial activity in live cells, TMRM quantifies the mitochondrial membrane potential, and Tom20 visualizes the mitochondrial mass in fixed cells. Therefore, the combined use of these three markers, namely MitoTracker, TMRM, and Tom20, provides a thorough evaluation of mitophagy by assessing mitochondrial function, membrane potential, and structural integrity.

To further validate these findings, we performed transmission electron microscopy (TEM) to analyze the mitochondrial number in the experimental groups. TEM analysis revealed a significant reduction in the number of mitochondria (19.4 ± 2.38) in the CCCP-treated group compared to the control (35.20 ± 4.90; *p* = 0.0096) (Figure 2c,d). Together, these results confirm the CCCP-induced mitophagy and a reduction in mitochondrial mass.

### 3.3. Induced Mitophagy Promotes Adult Cardiomyocyte Cell Cycle Re-Entry

**Figure 3:**
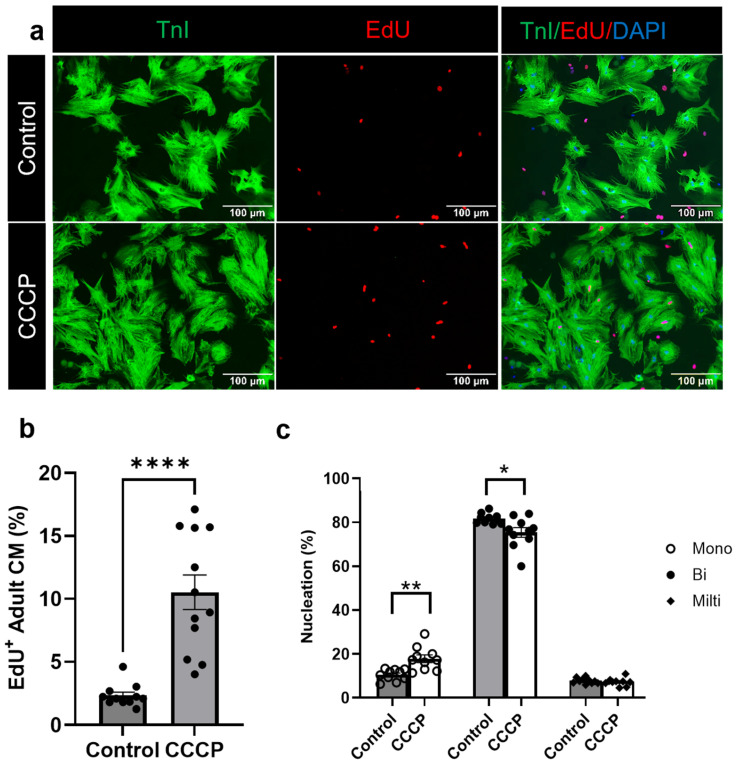
CCCP treatment leads to adult CM cell cycle progression: (**a**,**b**) Representative immunofluorescent images showing a higher number of EdU^+^ adult CMs in the CCCP-treated group compared to the control. (**c**) Bar graph representing the percentage distribution of mono-, bi-, and multinucleated adult CMs in the CCCP-treated group *versus* control. Scale bar = 100 µm. Results are presented as mean ± SE; * = *p* ≤ 0.05; ** = *p* ≤ 0.01; **** = *p* ≤ 0.0001. Statistical significance was determined using an unpaired *t*-test, with *p* ≤ 0.05 considered statistically significant. A minimum of three images per group per trial were analyzed, and the experiment was repeated at least three times.

After observing a significant induction in the mitophagy, we next analyzed the adult CM proliferation in parkin-overexpressing adult CMs following CCCP treatment. The in vitro proliferation assay revealed a significant increase in adult CM cell cycle re-entry following parkin and CCCP-induced mitophagy. Notably, we observed a marked increase in the DNA synthesis marker of EdU-positive adult CMs in the CCCP-treated group (10.52 ± 0.14%) compared to the control group (2.32 ± 0.27%, *p* < 0.0001) (Figure 3a,b). For the clearer visualization and better separation of fluorescence channels, representative images for EdU staining are also rendered in a magenta/cyan format (Supplementary Figure S6).

A further analysis of CM nucleation revealed a significant increase in mono-nucleated adult CMs in the CCCP-treated group (17.82 ± 1.71) compared to the controls (10.46 ± 0.80, *p* = 0.001). In contrast, the proportion of bi-nucleated adult CMs was significantly reduced in the CCCP-treated group (75.35 ± 2.21) compared to the controls (81.68 ± 0.73, *p* < 0.01) (Figure 3c). No significant differences were observed in the multinucleated adult CM populations between experimental groups.

**Figure 4:**
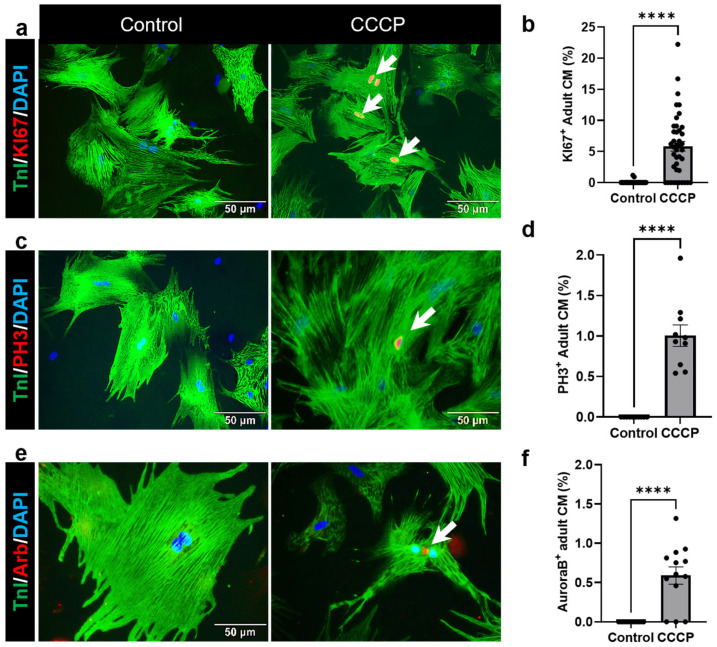
Increased proliferation and mitotic activity in CCCP-treated adult CM: Representative immunofluorescent images showing adult CMs positive for (**a**) KI67, (**c**) PH3, and (**e**) AuroraB in the CCCP-treated group compared to the control. The corresponding bar graphs quantify the proportion of (**b**) KI67-, (**d**) PH3-, and (**f**) AuroraB-positive CMs in the CCCP-treated group compared to the control. White arrow indicates the adult cardiomyocyte positive for KI67, PH3, or AuroraB. Scale bar = 50 µm. Data are expressed as mean ± SE; **** = *p* ≤ 0.0001. Statistical significance was determined using an unpaired *t*-test, with *p* ≤ 0.05 considered significant. A minimum of three images per group per trial were analyzed, and the experiment was repeated at least three times.

In addition to EdU, we evaluated the expression of other cell cycle markers, including the DNA synthesis marker KI67, the mitosis marker PH3, and the cytokinesis marker Aurora B. A significant increase in KI67-positive adult CMs was observed in the CCCP-treated group (5.84 ± 0.79%) compared to the controls (0.074 ± 0.042%, *p* < 0.0001) (Figure 4a,b). Similarly, our results demonstrated a significant increase in PH3-positive adult CMs in the CCCP-treated group (1.004 ± 0.13, *p* < 0.0001), whereas no PH3-positive CMs were detected in the control group (Figure 4c,d). Furthermore, Aurora B staining revealed a significant increase in adult CMs at the cytokinesis stage in the CCCP-treated group (0.59 ± 0.11, *p* < 0.0001), while no Aurora B-positive CMs were observed in the control group (Figure 4e,f). For the clearer visualization and better separation of fluorescence channels, representative images for KI67, PH3, and AuroraB staining are also rendered in a magenta/cyan format (Supplementary Figures S7–S9, respectively).

Overall, these results provide strong evidence for the activation of the cell cycle in adult CMs following mitophagy induction, highlighting the potential role of mitophagy in adult CM senescence.

### 3.4. Parkin and CCCP-Mediated Induced Mitophagy Results in Reduced Oxidative Stress in Adult Cardiomyocyte

To assess oxidative stress following parkin- and CCCP-mediated mitophagy in adult CMs, we employed a combination of three distinct markers: DHR123, CellROX, and VDAC staining. DHR123 is a well-established marker for mitochondrial ROS, allowing us to specifically quantify mitochondrial-generated ROS. CellROX serves as a broad-spectrum indicator for overall cellular oxidative stress, while VDAC staining was used to assess mitochondrial integrity, as it is localized to the mitochondrial outer membrane.

**Figure 5:**
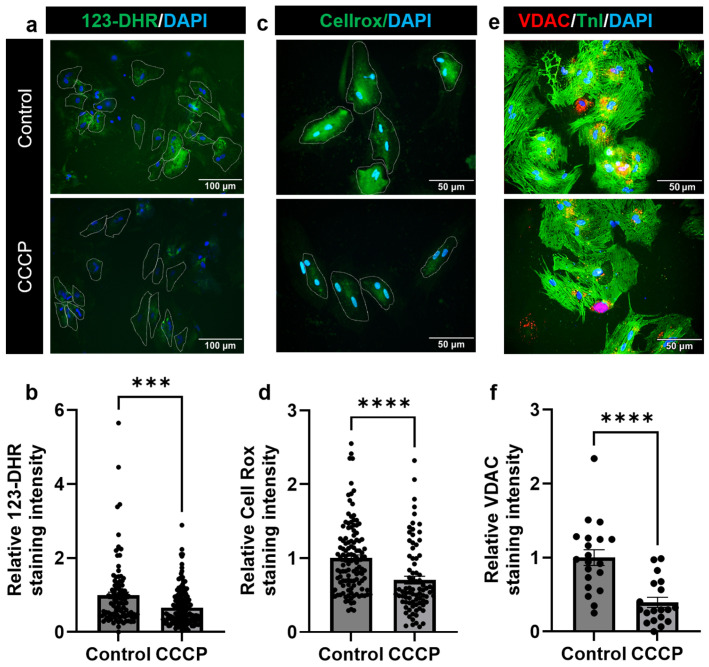
CCCP treatment reduces ROS in adult CM. (**a**,**b**) Representative immunofluorescent images showing 123-DHR staining, along with a bar graph quantifying the fluorescence intensity of 123-DHR in the CCCP-treated group compared to control. (**c**,**d**) Representative immunofluorescent images for CellROX and the corresponding bar graph quantifying the fluorescence intensity of CellROX in the CCCP-treated group *versus* control. (**e**,**f**) Representative immunofluorescent images for VDAC, along with a bar graph quantifying VDAC fluorescence intensity in the CCCP-treated group compared to the control. Scale bar = 50 µm or as indicated. Data are presented as mean ± SE; *** = *p* ≤ 0.0002; **** = *p* ≤ 0.0001. Statistical significance was determined using an unpaired *t*-test, with *p* ≤ 0.05 considered significant. A minimum of 30 cells per experimental group were analyzed per experiment, and the experiment was repeated at least three times. White dotted lines delineate the boundaries of individual CMs.

The combined use of these three markers provided a thorough and multi-faceted analysis of oxidative stress in response to CCCP-induced mitophagy. We observed a significant decrease in all oxidative stress markers in CCCP-treated adult CMs compared to control cells. Specifically, the DHR123 fluorescence intensity was significantly reduced in the CCCP-treated group (0.69 ± 0.04) compared to the control (1 ± 0.09; *p* = 0.0002), indicating a reduction in mitochondrial ROS (Figure 5a,b). CellROX staining also showed a marked decrease in cellular oxidative stress in the CCCP-treated group (0.70 ± 0.05) when compared to the control (1 ± 0.43; *p* < 0.0001) (Figure 5c,d). Similarly, the intensity of VDAC staining, which reflects mitochondrial integrity, was significantly reduced (0.39 ± 0.07) in CCCP-treated CMs compared to the control (1 ± 0.10; *p* < 0.0001) (Figure 5e,f). These results suggest that CCCP treatment not only reduces mitochondrial ROS but also decreases general cellular oxidative stress and mitochondrial dysfunction.

**Figure 6:**
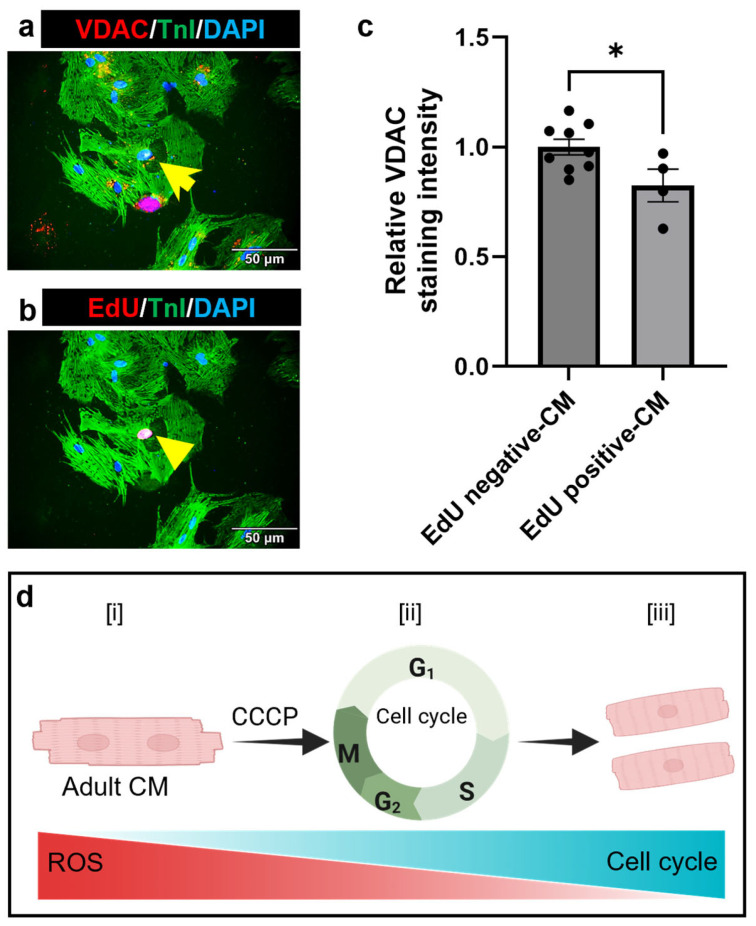
Reduced oxidative stress in recycling adult CM. (**a**) Representative image of an adult CM exhibiting lower VDAC intensity compared to neighboring cells, as indicated by the yellow arrow. (**b**) The same CM is positive for the cell cycle marker EdU, as indicated by the yellow arrowhead. Scale bar = 50 µm. (**c**) The bar graph demonstrates the relative VDAC level in proliferating *versus* non-proliferating CMs in the parkin-transfected and CCCP-treated group. Data are presented as mean ± SE; * = *p* ≤ 0.05. Statistical significance was determined using an unpaired *t*-test, with *p* ≤ 0.05 considered significant. A minimum four images per condition from one experiment were analyzed. (**d**) Graphical representation illustrating that the reduction in oxidative stress may facilitate cell cycle progression in adult CMs.

Additionally, we observed an intriguing relationship between oxidative stress and cell cycle re-entry. Despite CCCP-treated cells exhibiting lower VDAC levels compared to the control, notably, the proliferating adult CMs, identified as EdU-positive cells (indicated by yellow arrowhead), displayed even lower VDAC levels (17.5% lower, *p* = 0.03) than the non-proliferating CMs (indicated by yellow arrow) within the same CCCP-treated group (Figure 6a–c). This finding is particularly noteworthy as it suggests that the reduction in oxidative stress may play a critical role in promoting cell cycle re-entry and mitigating the senescence-associated phenotype of adult CMs (Figure 6d). Taken together, these results demonstrate that CCCP-mediated mitophagy leads to a significant reduction in both mitochondrial and cellular oxidative stress, which may contribute to the observed increase in cell cycle re-entry in adult CMs.

## 4. Discussion

The present study demonstrates the activation of adult CM proliferation by induced mitophagy, accompanied by a significant decrease in oxidative stress. The post-natal oxygen-rich environment, along with the metabolic shift from glycolysis to oxidative phosphorylation, plays a critical role in inducing cell cycle exit and contributing to the senescent nature of adult CMs [6,35]. Interestingly, studies in zebrafish, which possess lifelong cardiac regenerative capacity, have highlighted a hypoxic signature as a key feature of their CM proliferation [3,12,13,36]. Consistent with this, recent research in adult mice indicates that hypoxic exposure can suppress oxidative stress and DNA damage responses, thereby promoting CM proliferation and enhancing cardiac repair following infarction [21]. In parallel, mitophagy has emerged as a critical regulatory process in cellular stress responses. For instance, mitophagy induction in fibroblasts has been shown to reduce oxidative stress and DNA damage, thereby enhancing the proliferative capacity [23]. Additional studies have further demonstrated that upregulated mitochondrial clearance through autophagy alleviates the accumulation of dysfunctional mitochondria, reduces ROS, and supports cardiomyocyte survival and function [37,38]. Notably, Zhang et al. reported that ROS accumulation secondary to mitochondrial dysfunction induces DNA damage and arrests the CM cell cycle [39]. Importantly, the pharmacological inhibition of ROS was sufficient to restore cell cycle progression, reinforcing the causal link between oxidative stress and cell cycle exit in adult cardiomyocytes [39]. While oxidative stress-induced cell cycle arrest in adult CMs is well stated, the potential for reduced oxidative stress through mitophagy to promote CM proliferation remains largely unexplored. Therefore, this study aimed to investigate whether the reduction in oxidative stress via parkin- and CCCP-mediated induced mitophagy could facilitate adult CM proliferation.

In this study, we utilized a parkin-CCCP-mediated approach to induce mitophagy in the adult rat CMs. CCCP, a mitochondrial uncoupler, induces membrane depolarization, while parkin, an E3 ubiquitin ligase, facilitates the ubiquitination and proteasomal degradation of damaged mitochondria, a process known as mitophagy [23,31]. The effectiveness of CCCP treatment is dose- and time-dependent, varying with cell type and treatment duration [40,41,42]. Importantly, long-term interventions are critical for investigating adult CM proliferation, as the reprogramming of the senescent phenotype requires extended periods, as we have previously shown. Unlike induced pluripotent stem cell (iPSC)-derived CMs or other inherently proliferative cell types, adult CMs require extended time to re-establish the molecular machinery necessary for cell cycle re-entry. Consistent with this, we and others have shown that proliferative responses in adult CMs typically peak around day 7 following pro-proliferative interventions [33,43]. A study utilizing the p38 MAP kinase inhibitor SB203580 extended treatment to 12 days before assessing proliferation, highlighting the critical role of sustained intervention in enabling cell cycle reactivation in adult CMs [44,45]. Taking these temporal dynamics into account, we chose day 7 post-treatment as the optimal time point to evaluate both CM proliferation and oxidative stress in the present study.

In our study, we identified 3 µM as the optimal dose of CCCP for the long-term treatment of adult CMs. Additionally, we observed that CCCP concentrations below 2 μM were insufficient to induce significant cardiomyocyte proliferation, while concentrations exceeding 4 μM led to increased cytotoxicity and cell death. Our analysis reveals a clear reduction in the mitochondrial mass in live adult CMs following CCCP treatment, as indicated by the membrane potential-dependent probes MitoTracker and TMRM. This finding was further supported by TOM20 labeling, a marker of the mitochondrial outer membrane. Consistent with these observations, TEM analysis showed a significant decrease in the mitochondrial number, providing the ultrastructural confirmation of CCCP-induced mitophagy.

Next, to determine whether mitophagy induction promotes adult CM proliferation, we undertook a comprehensive assessment of cell cycle activity following CCCP and parkin treatment. Unlike non-myocytes or inherently proliferative cell types, evaluating proliferation in adult CMs is particularly challenging due to their terminally differentiated phenotype and restricted regenerative capacity. Accordingly, we employed a multi-parametric approach, as reliance on a single marker is insufficient to capture the complexity of CM cell cycle dynamics. This included markers spanning distinct stages of the cell cycle, complemented by nucleation analysis to provide structural insight.

Specifically, EdU incorporation and Ki67 expression were used to assess DNA synthesis and overall cell cycle activation, while progression through mitosis and cytokinesis was evaluated via PH3 and Aurora B kinase, respectively. In parallel, we assessed the CM nucleation status, given that adult CMs are predominantly binucleated due to postnatal karyokinesis without cytokinesis [46,47]. An increase in mononucleated CMs is considered indicative of cell cycle re-entry and the potential completion of cytokinesis. Together, this integrated analysis revealed a significant increase in CM cell cycle progression following mitophagy activation, supporting the notion that the targeted enhancement of mitochondrial quality control may serve as a viable strategy to promote regenerative activity in the adult heart.

Moreover, we observed a decrease in oxidative stress in the CCCP-treated group, as evidenced by various in vitro assays, including CellROX, DHR123, and VDAC analysis. Notably, an in-depth analysis revealed that proliferating adult CMs exhibited even lower oxidative stress levels compared to their surrounding non-proliferating CMs.

Our results are consistent with previous reports suggesting the existence of a distinct population of CMs that respond more effectively to external proliferative interventions [42,48,49]. Furthermore, our study substantiates that an increased number of mitochondria in adult CMs contributes to the senescent phenotype of these cells by elevating oxidative stress. While our study highlights the role of oxidative stress in promoting the senescent phenotype of adult CMs, it also raises several important questions that warrant further investigation. Specifically, it is crucial to explore whether oxidative stress could serve as a marker for identifying CMs that are more responsive to proliferative interventions. Additionally, it remains essential to determine whether proliferating CMs retain a hypoxic phenotype in contrast to their non-proliferating counterparts. Furthermore, the DNA damage response in proliferating CMs following CCCP-induced mitophagy remains unanalyzed, as understanding these molecular mechanisms could shed light on how CMs achieve stress resilience and proliferation. These research directions could provide valuable insights into the regenerative potential of CMs and their response to therapeutic interventions.

Collectively, our findings offer compelling evidence that parkin- and CCCP-mediated mitophagy can induce adult CM cell cycle re-entry (Figure 6d). Our results suggest that certain adult CMs may possess a recycling phenotype, with an enhanced proliferative capacity in response to external stimuli, compared to their non-proliferating counterparts [22,50,51]. These proliferating CMs may exhibit distinct metabolic profiles, an area that warrants further exploration. Ultimately, understanding the characteristics of proliferating *versus* senescent CMs could be pivotal for developing targeted therapeutic interventions aimed at heart regeneration.

## Data Availability

The original contributions presented in this study are included in the article/Supplementary Material. Further inquiries can be directed to the corresponding author.

## Supplementary Materials

The following supporting information can be downloaded at: https://www.mdpi.com/article/10.3390/cells14120853/s1, Table S1: Adult Rat Cardiomyocyte Isolation Buffer Composition. Table S2: Details of the antibodies. Figure S1: Immunostaining images showing EdU-positive adult cardiomyocytes (CMs) treated with various concentrations of Rapamycin. (a) DMSO control; (b) Rapamycin 2 μM; (c) Rapamycin 4 μM; (d) Rapamycin 6 μM; (e) Rapamycin 10 μM. (f) Bar graph represents the quantification of EdU positive CMs among the study groups. CMs were identified by Troponin I staining (green), proliferating CMs by EdU incorporation (red), and nuclei by DAPI (blue). Scale bar: 100 μm. Data are presented as mean ± SE. ns = non-significant. Statistical significance was analyzed using one-way ANOVA. All experiments were conducted in triplicate. Figure S2: Immunostaining images showing EdU-positive adult cardiomyocytes (CMs) treated with various concentrations of Chloramphenicol. (a) DMSO control; (b) Chloramphenicol 2 μM; (c) Chloramphenicol 4 μM; (d) Chloramphenicol 6 μM; (e) Chloramphenicol 10 μM. (f) Bar graph represents the quantification of EdU positive CMs among the study groups. CMs were identified by Troponin I staining (green), proliferating CMs by EdU incorporation (red), and nuclei by DAPI (blue). Scale bar: 100 μm. Data are presented as mean ± SE. ns = non-significant; * = *p* < 0.05. Statistical significance was analyzed using one-way ANOVA. All experiments were conducted in triplicate. Figure S3: Immunostaining images showing EdU-positive adult cardiomyocytes (CMs) treated with 2 μM of Rapamycin (Rap) along with various concentrations (2-10 μM) of Chloramphenicol (Chl).(a) DMSO control; (b) Rapamycin (2 μM) + Chloramphenicol (2 μM); (c) Rapamycin (2 μM) + Chloramphenicol (4 μM); (d) Rapamycin (2 μM) + Chloramphenicol (6 μM); (e) Rapamycin (2 μM) + Chloramphenicol (10 μM). (f) Bar graph represents the quantification of EdU positive CMs among the study groups. CMs were identified by Troponin I staining (green), proliferating CMs by EdU incorporation (red), and nuclei by DAPI (blue). Scale bar: 100 μm. Data are presented as mean ± SE. ns = non-significant. Statistical significance was analyzed using one-way ANOVA. All experiments were conducted in triplicate. Figure S4: (a) Bar graph represents the quantification of EdU positive CMs after varying concentrations of CCCM treatment *versus* control. (b) bar graph represents the quantification of EdU positive CMs after CCCM (3 μM) and Chloramphenicol (2 μM) treatment, when compared to control. Data are presented as mean ± SE. ns = non-significant; ** = *p* = 0.002; *** = *p* < 0.0001. Statistical significance was analyzed using one-way ANOVA. All experiments were conducted in triplicate. Figure S5: Representative immunofluorescent images showing the higher Tom20 expression in control (a), when compared to CCCP treated group (b). Scale bar = 50 μM. Cayn color = troponin; Magenta color = VDAC. Figure S6: Representative immunofluorescent images showing the higher EdU positive cardiomyocytes in CCCP treated group (b), when compared to control (a). Scale bar = 100 μM. Cayn color = troponin; Magenta color = EdU. Figure S7: Representative immunofluorescent images showing the higher KI67 positive cardiomyocytes in CCCP treated group (b), when compared to control (a). Scale bar = 50 μM. Cayn color = troponin; Magenta color = KI67. Figure S8: Representative immunofluorescent images showing the higher PH3 positive cardiomyocytes in CCCP treated group (b), when compared to control (a). Scale bar = 50 μM. Cayn color = troponin; Magenta color = PH3. Figure S9: Representative immunofluorescent images showing the higher AuroraB positive cardiomyocytes in CCCP treated group (b), when compared to control (a). Scale bar = 100 μM. Cayn color= troponin; Magenta color = AuroraB.
