# Supplementary material for: Induced Mitophagy Promotes Cell Cycle Re-Entry in Adult Cardiomyocytes
Source: Cells. 2025 Jun 6;14(12):853. doi: 10.3390/cells14120853 (PMC12190997; doi:10.3390/cells14120853)

Induced mitophagy promotes cell cycle re-entry in adult cardiomyocytes.

Table S1: Adult Rat Cardiomyocyte Isolation Buffer Composition

**10X KHB Stock Solution (Total volume= 1L)**	**Reagent**	**Molarity (mM)**
	NaCl	1180
	KCl	48
	HEPES	250
	$MgSO_4$	12.5
	K_2HPO_4	12.5
**KHB Solution, 500 mL**	**Reagent**	**Amount**
	10X KHB	50 mL
	Glucose	0.99 g
	Taurine	0.31 g
	Bring volume to 500mL with H_2O, and pH should be ~7.35	
**Solution A**	**Reagent**	**Amount**
	KHB solution	500 mL (10 mM)
	BDM	0.5 g
	Oxygenate with 100% O_2 and warm to 37°C	
**Solution B, 50mL**	**Reagent**	**Amount**
	Solution A	50 mL
	BSA	0.5 g
	0.1 M $CaCl_2$ (Ca^{++}=0.1 mM)	50 µL
**Solution E, 50mL**	**Reagent**	**Amount**
	Solution A	50 mL
	BSA	0.05 g
	Collagenase type II (263 units/mg)	35 mg
	Hyaluronidase (Type I-S)	10 mg
	0.1 M $CaCl_2$ stock	12.5 µL
	Mix well	
**CaCl2 Stock, 0.1M**	**Reagent**	**Amount**
	$CaCl_2$	7.35 g
	H_2O	500 mL
	Store at 4°C	

Table S2: Details of the antibodies

	Antibody	Catalog #	Company
1	Rabbit anti-aurora B	A5102	Sigma-Aldrich
2	Mouse anti- cardiac troponin-T	MA5-12960	Thermo Scientific
3	Rabbit anti-cardiac troponin-I	sc-15368	Santa Cruz Biotechnology
4	Mouse-anti-Histone H3	Ab6002	Abcam
5	Mouse anti-Ki67	550609	BD Pharmingen
6	Mouse-Anti-TOMM20	ZMS1118	Sigma-Aldrich
7	Goat anti-mouse Alexa fluor 488	A11029	Life Technologies
8	Goat anti-mouse Alexa fluor 594	A11005	Life Technologies
9	Goat anti-rabbit Alexa fluor 488	A11008	Life Technologies
10	Goat anti-rabbit Alexa fluor 594	A11037	Life Technologies

Figure S1

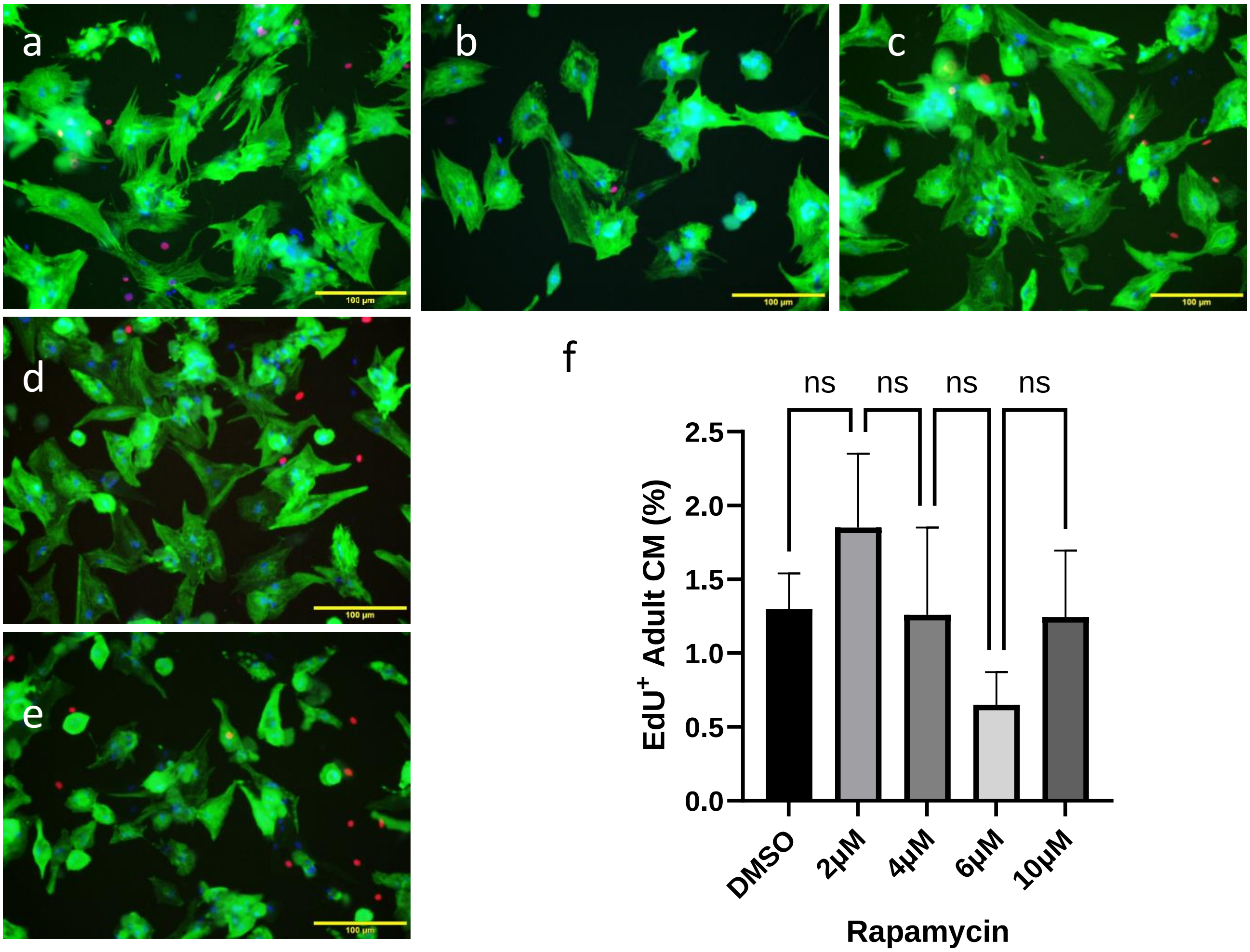
a
b
c
d
e
f
100 µm
ns
ns
ns
ns
2.5
2.0
1.5
1.0
0.5
0.0
EdU+ Adult CM (%)
DMSO
2µM
4µM
6µM
10µM
Rapamycin

Figure S2

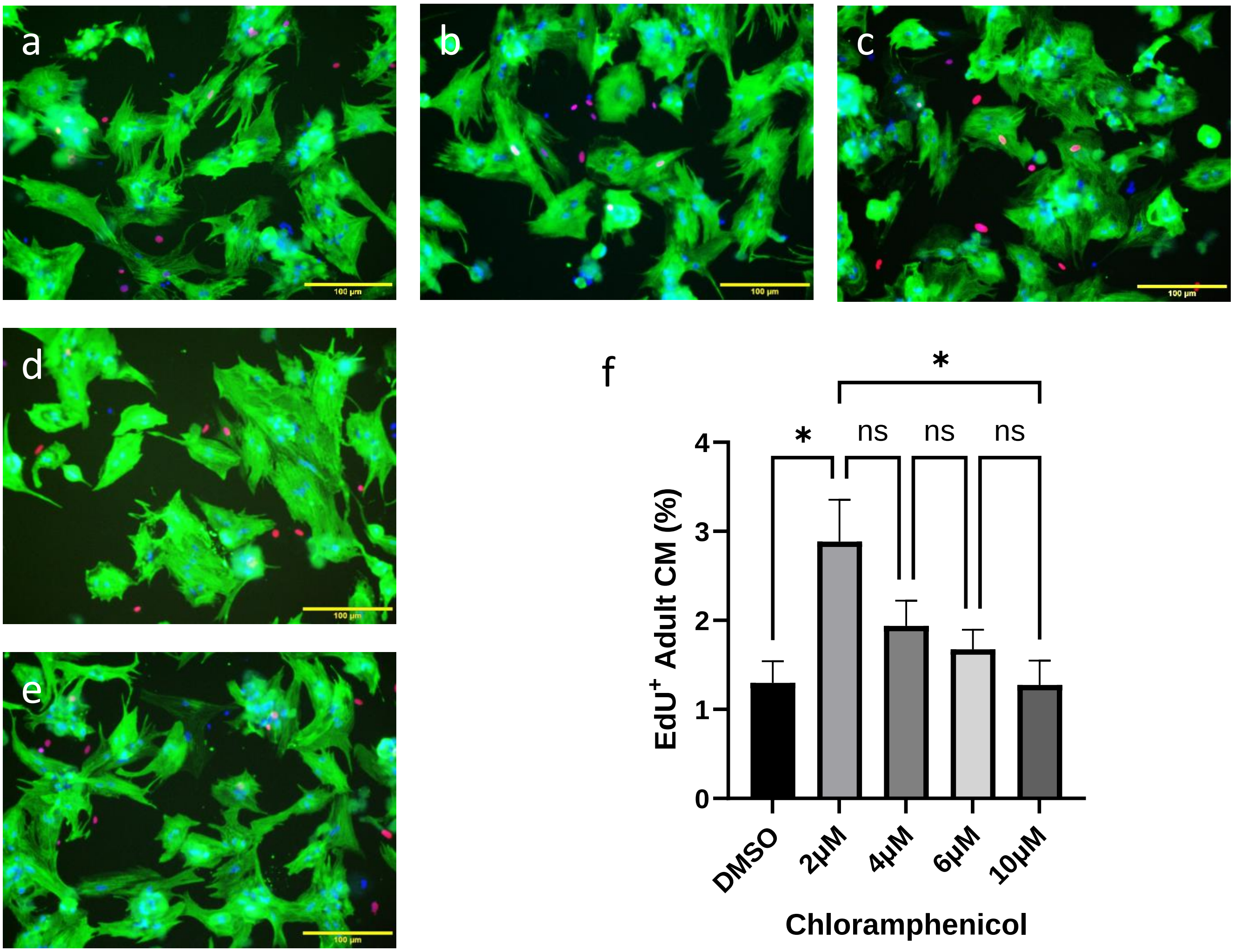
a
b
c
d
e
f
100 µm
EdU+ Adult CM (%)
DMSO
2µM
4µM
6µM
10µM
Chloramphenicol
ns
*

Figure S3

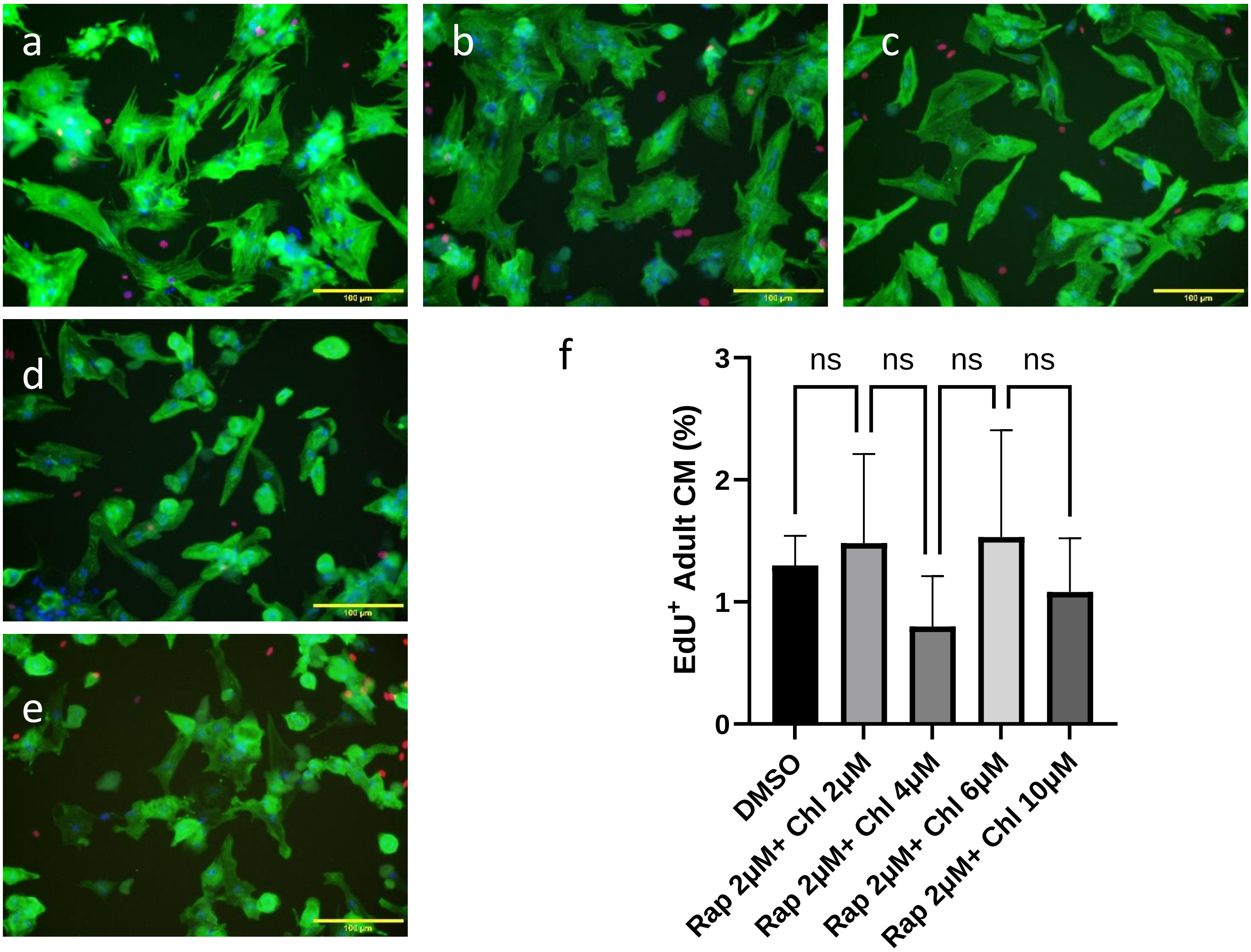

a
100 µm
b
100 µm
c
100 µm
d
100 µm
e
100 µm
f
EdU+ Adult CM (%)
0
1
2
3
ns
ns
ns
ns
DMSO
Rap 2µM+ Chl 2µM
Rap 2µM+ Chl 4µM
Rap 2µM+ Chl 6µM
Rap 2µM+ Chl 10µM

Figure S4

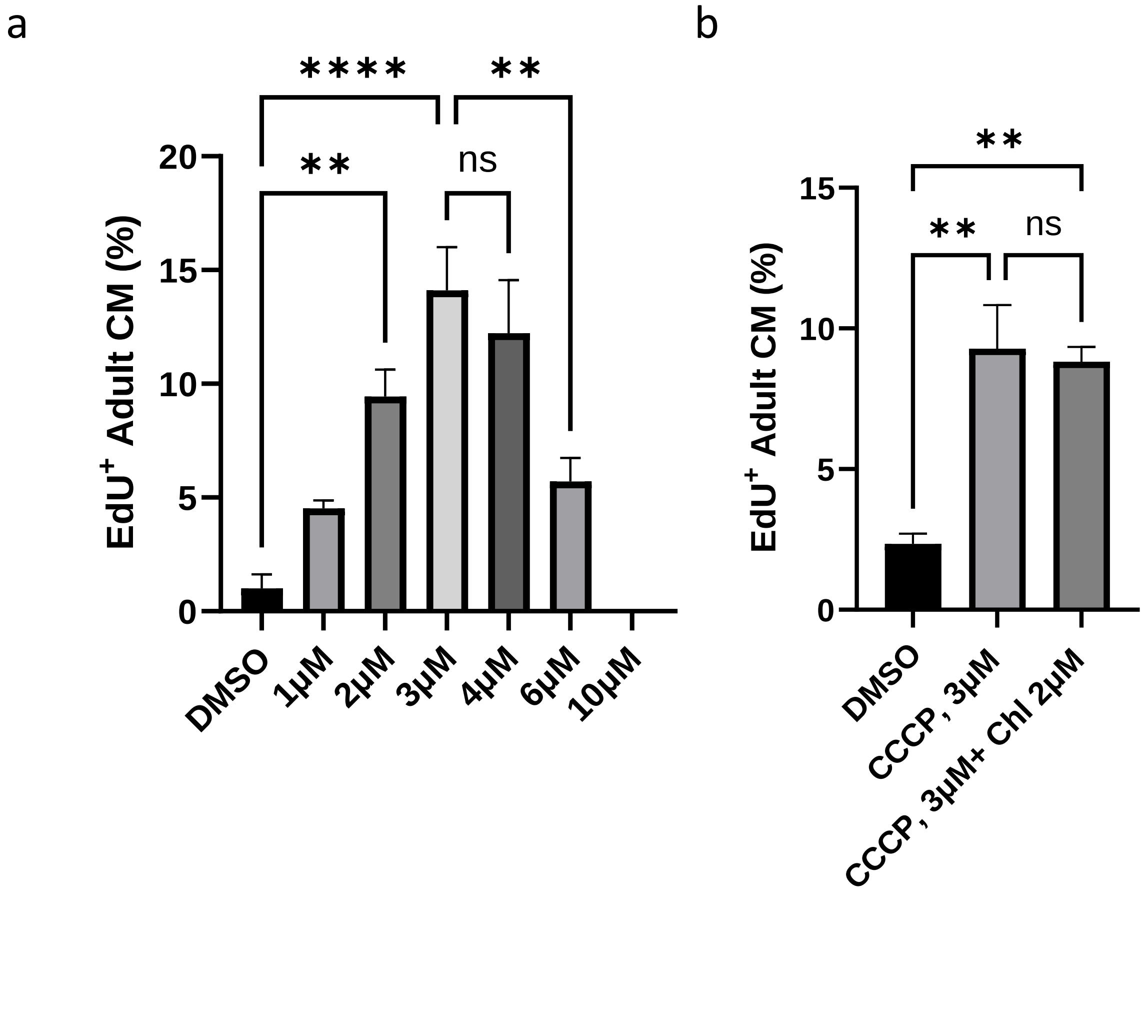
a
****
**
**
ns
20
15
10
5
0
EdU+ Adult CM (%)
DMSO
1μM
2μM
3μM
4μM
6μM
10μM
b
**
**
ns
15
10
5
0
EdU+ Adult CM (%)
DMSO
CCCP, 3μM
CCCP, 3μM+ Chl 2μM

Figure S5

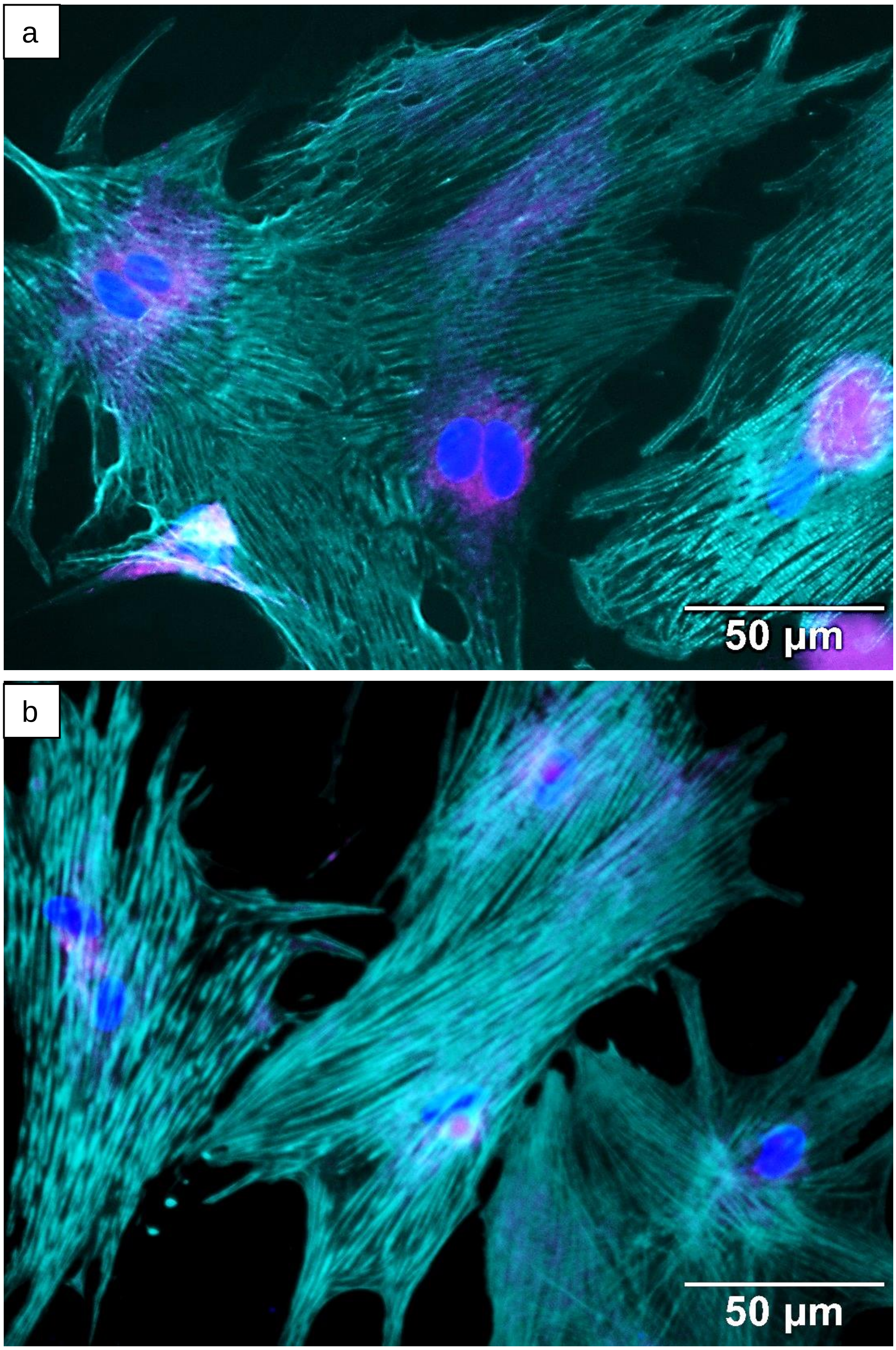

Figure S6

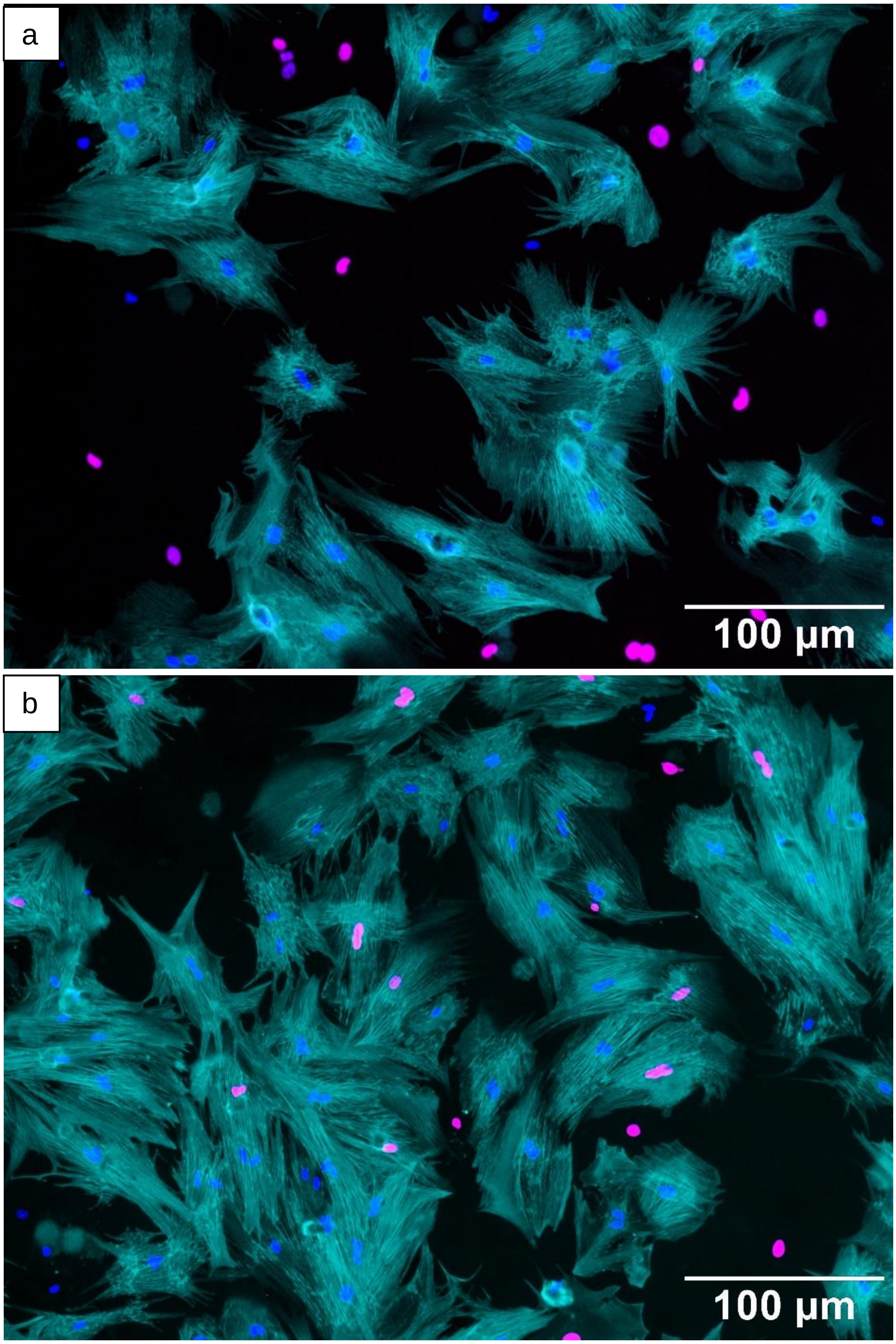

Figure S7

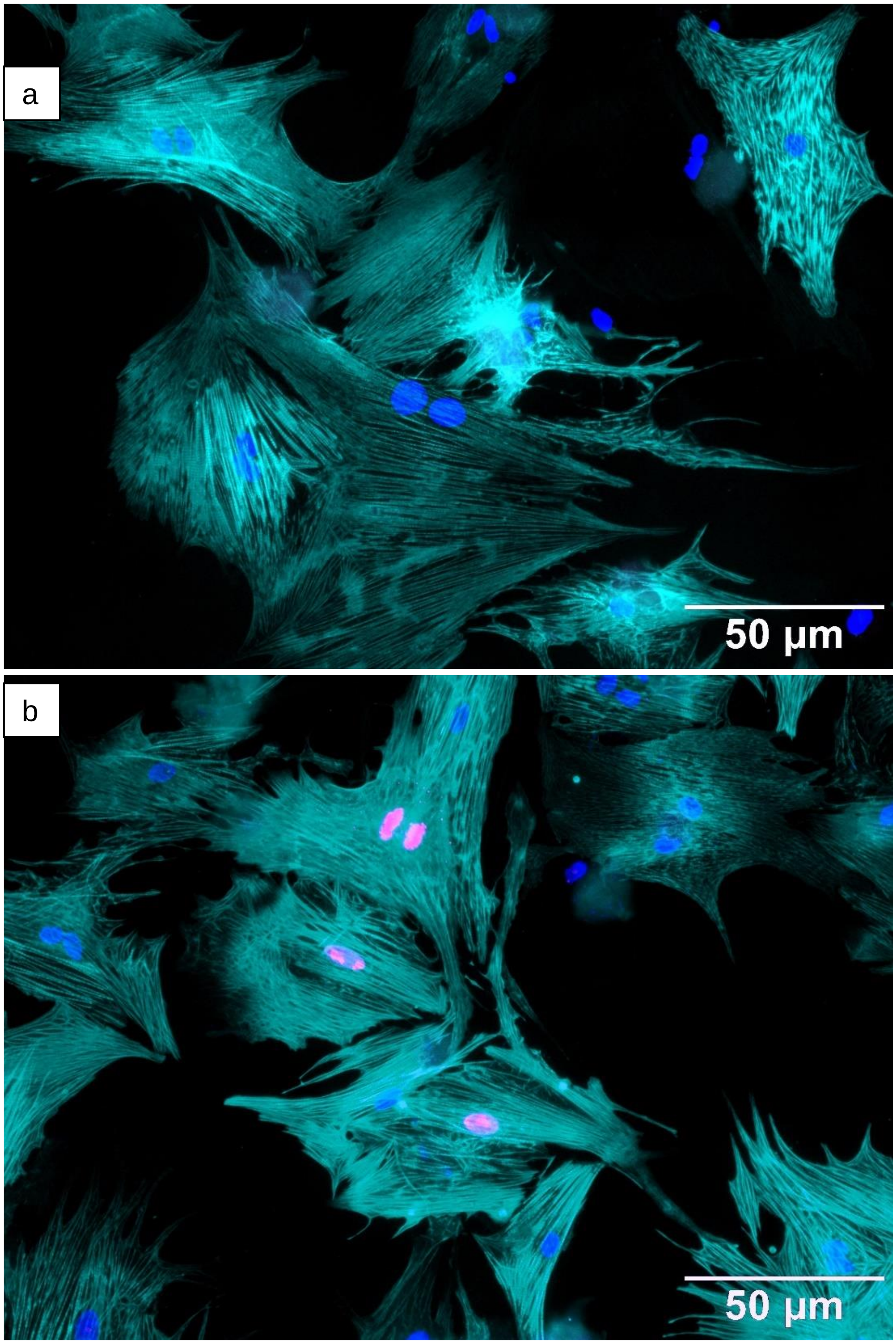

Figure S8

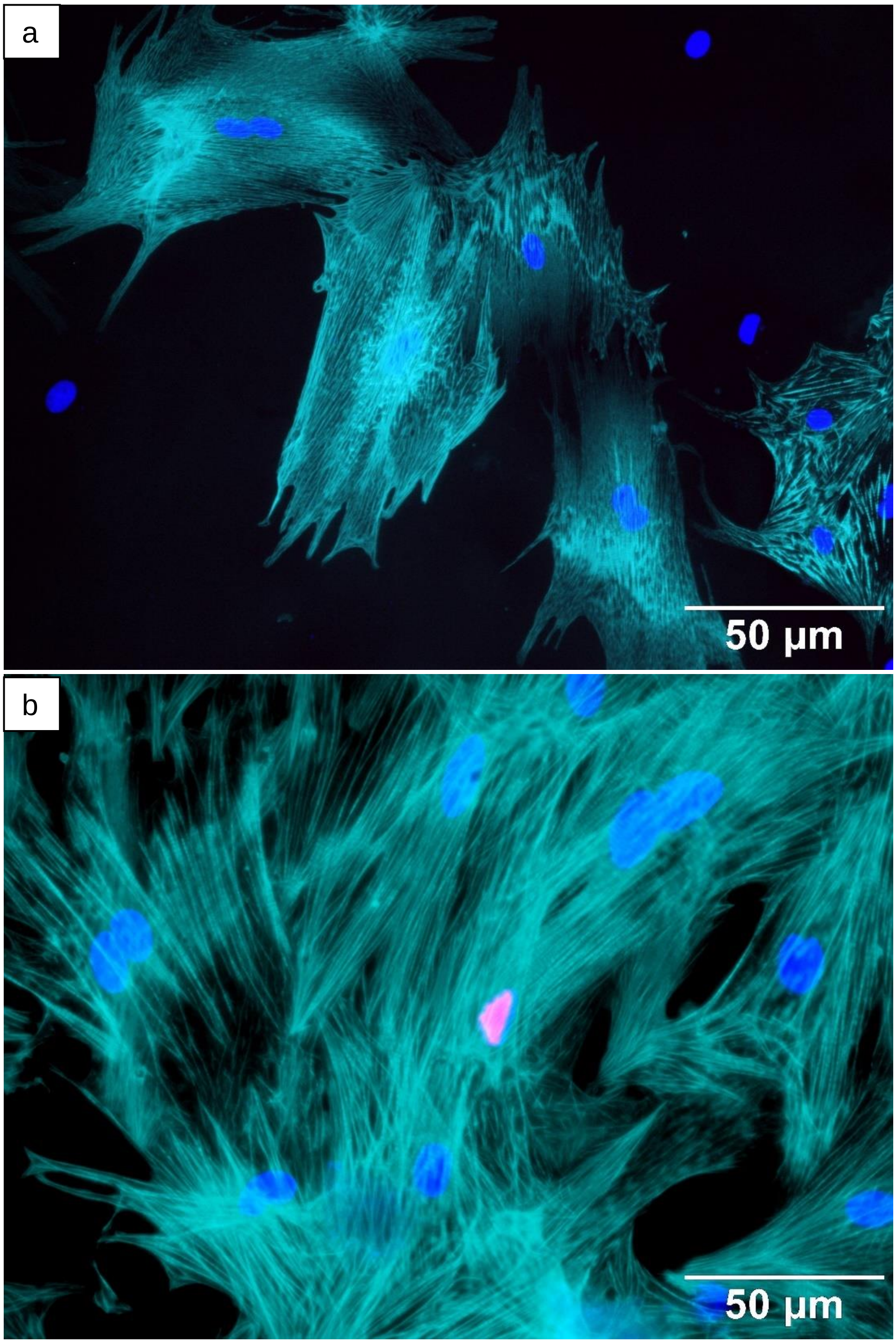

Figure S9

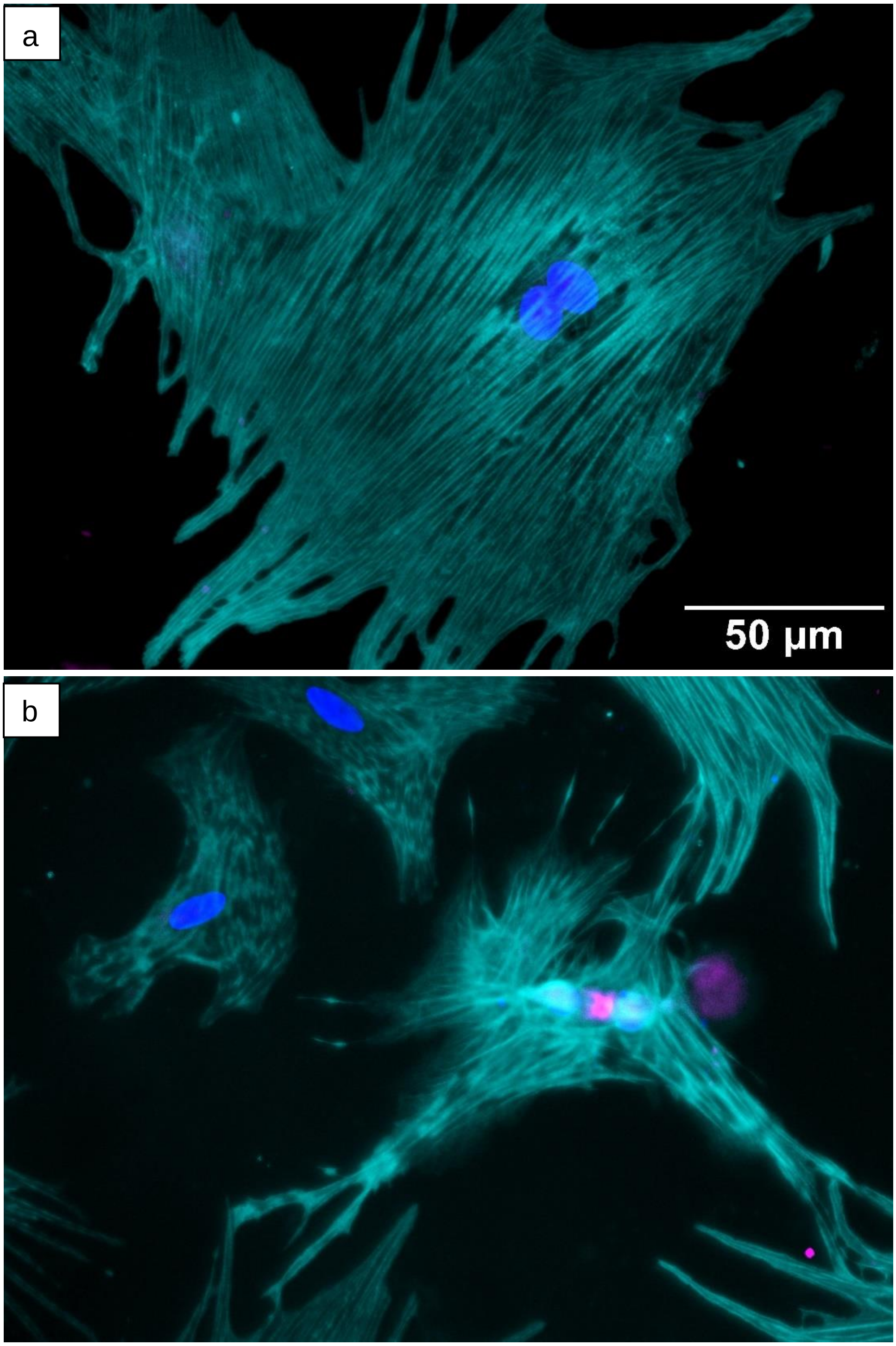

Supplement: Supplementary file 1 [file cells-14-00853-s001.zip › cells-3563217-supplementary.pdf]
